# Supplementary material for: The regulatory light chain mediates inactivation of myosin motors during active shortening of cardiac muscle
Source: Nat Commun. 2021 Sep 6;12:5272. doi: 10.1038/s41467-021-25601-8 (PMC8421338; doi:10.1038/s41467-021-25601-8)
Supplement: Supplementary file 1 — Supplementary Information [file 41467_2021_25601_MOESM1_ESM.pdf]

Supplementary Information

The regulatory light chain mediates inactivation of myosin motors during active shortening of cardiac muscle

Thomas Kampourakis* and Malcolm Irving

Randall Centre for Cell and Molecular Biophysics, and British Heart Foundation Centre of Research Excellence, King's College London, London, SE1 1UL, United Kingdom

Short Title: Active shortening switches off cardiac myosin

*Corresponding author: Thomas Kampourakis (thomas.kampourakis@kcl.ac.uk)

Supplementary Figures and Figure Legends

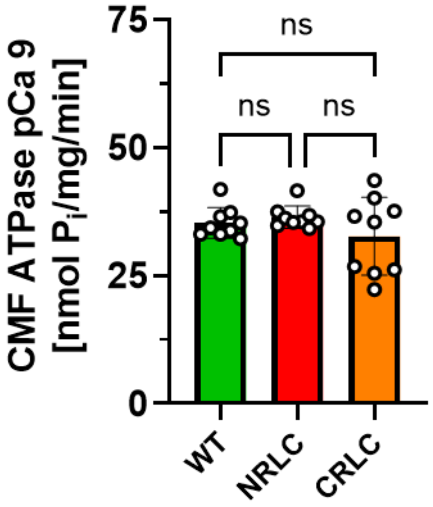

Supplementary Figure 1. Effect of wildtype RLC (WT, green), NRLC (red) and CRLC probe (orange) exchange on cardiac myofibrillar ATPase activity under relaxing conditions (pCa 9). Statistical significance of differences was assessed with a one-way ANOVA followed by Tukey's post-hoc test: ns – not significant. Means ± s.e.m. Pooled data from n=3 different cardiac myofibril preparations. Source data are provided as a Source Data file.

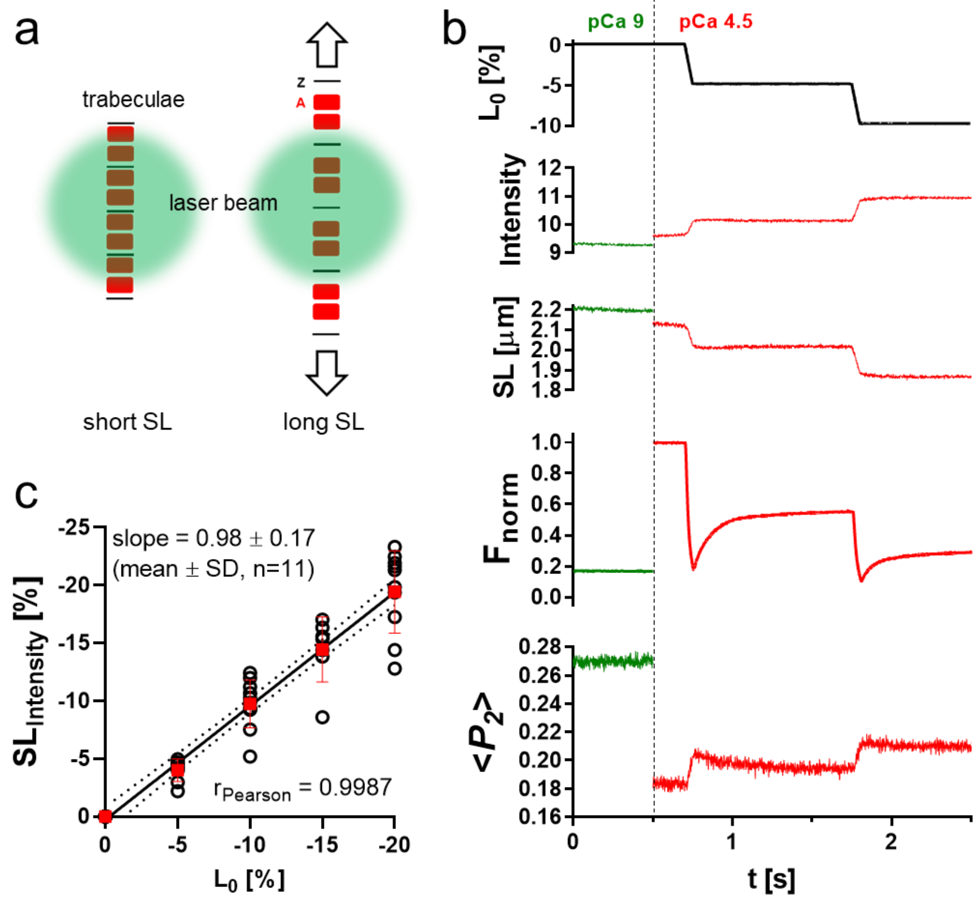

Supplementary Figure 2. Estimating sarcomere length (SL) in the sarcomeres in the measuring beam using fluorescence intensity. (a) Cartoon representation showing the principle of the measurements. At short SL (left) more sarcomeres (red) are in the laser beam (green) than at long SL (right), resulting in decreased fluorescence intensity with increased sarcomere length. (b) Example traces of trabeculae length, total fibre intensity, calculated sarcomere length, normalized force and $<P_2>$ measured from BSR-cRLC-E exchanged trabecula in relaxing (green, pCa 9) and activating conditions (red, pCa 4.5). (c) Relationship between sarcomere length and muscle length in fully calcium activated trabeculae. Data pooled from n=11 trabeculae. Continuous and dotted lines represent linear regression to data points and 95% confidence interval, respectively, with a slope of 0.98 ± 0.05 (mean ± 95%CI) and Y-intercept of 0.28 ± 0.56 (mean ± 95%CI). Pearson correlation coefficient (r) is shown at bottom right. Red squares indicate calculated average SL changes and standard deviations. Source data are provided as a Source Data file.

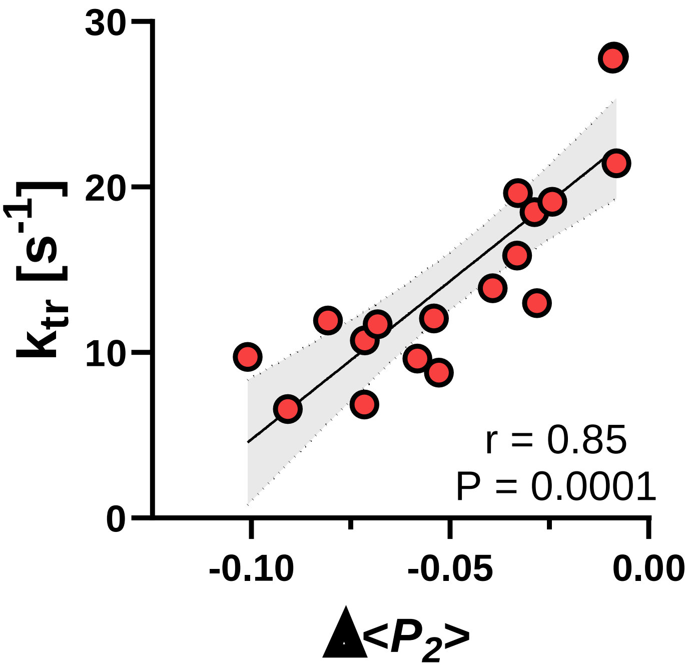

Supplementary Figure 3. Plot of <P_2> change (Δ<P_2>) of NRLC after the length step versus rate of force development k_{tr} (pooled data from n=5 trabeculae). Line represents linear regression to data points with a slope of 191.3 ± 31.5 s^{-1} Δ<P_2>$^{-1}$ and Y-intercept of 23.89 ± 1.75 s^{-1} (means ± 95%CI). Grey shaded areas indicate 95% confidence interval. Pearson correlation coefficient (r) and p-value for a two-tailed student's t-test are shown on the bottom right. Source data are provided as a Source Data file.

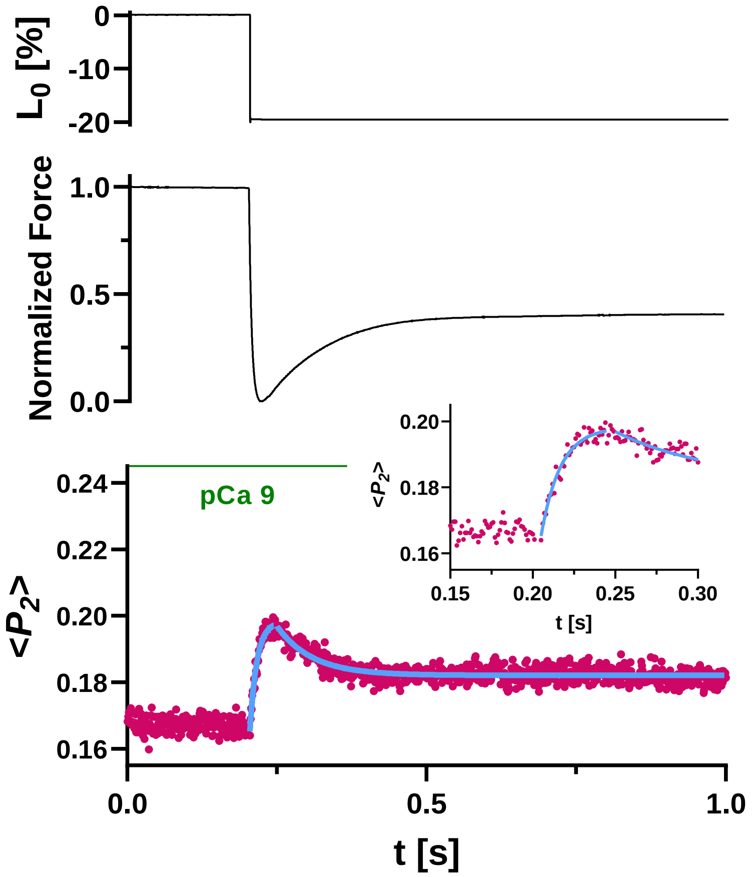

Supplementary Figure 4. <*P*$_2$> response measured from the cTnC E-helix probe (BR-cTnC-E) in the thin filaments during 20% L_0 shortening step of Ca^{2+}-activated ventricular trabeculae. Upper panel: muscle length; middle panel: force; lower panel: <*P*$_2$>. The relaxed value (pCa 9) of <*P*$_2$> from the cTnC E-helix probe is indicated by a green line. Inset: <*P*$_2$> response from the cTnC E-helix probe to shortening on an extended time scale. Single exponential fits are shown in cyan. Source data are provided as a Source Data file.

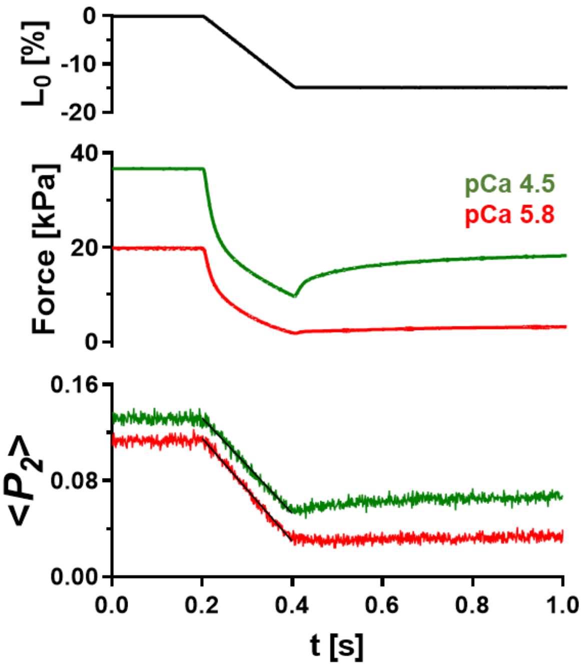

Supplementary Figure 5. Effect of [Ca^{2+}] on sarcomere-length dependence of <P_2> from the BC-helix probe. Top: muscle length, middle: normalized force, bottom: <P_2>. Black dotted lines in (B) represent linear fits to data points with slopes of -0.04 and -0.042 <P_2>·s^{-1} for pCa 4.5 and 5.8, respectively. Source data are provided as a Source Data file.

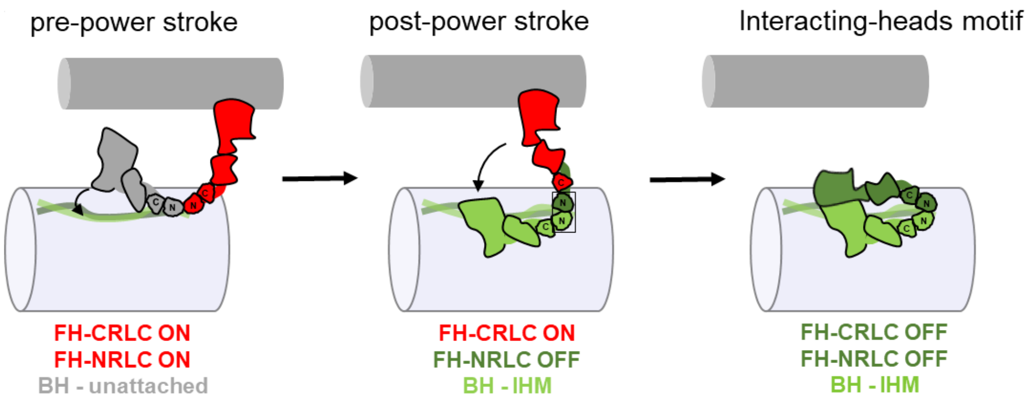

Supplementary Figure 6. Alternative model for the effect of shortening on myosin head conformation in cardiac muscle. The working stroke of the actin-attached free head (red) forces the unattached blocked head (grey) into the myosin tail-bound OFF state (post-power stroke), followed by detachment of the free head form actin and the formation of the interacting heads motif.

Supplementary Information Table and Table Legends

Supplementary Table 1. Summary of force-pCa and <P_2>-pCa paramaters, and rate constants (k) of force and <P_2> redevelopment after slack/strecth protocol for BSR-cRLC-BC and BSR-cRLC-E exchanged trabeculae.

	BSR-cRLC-BC		BSR-cRLC-E	
	Force	<P_2>	Force	<P_2>
F_{min} [kPa]	0.2 ± 0.1	-	0.2 ± 0.1	-
F_{max} [kPa]	28.9 ± 1.4	-	37.4 ± 3.1	-
<P_2>$_{min}$	-	-0.018 ±0.001	-	0.0742 ± 0.007
<P_2>$_{max}$	-	0.076 ± 0.015	-	0.171 ± 0.006
pCa_{50}	5.56 ± 0.01	5.61 ± 0.01*	5.56 ± 0.01	5.56 ± 0.01^{ns}
n_H	5.63 ± 0.52	8.13 ± 0.80*	6.85 ± 0.65	8.41 ± 0.66^{ns}
k [s-1]	12.0 ± 0.8	20.6 ± 1.7**	11.8 ± 0.8	14.6 ± 1.0^{ns}

Values indicate means ± s.e.m. Statistical significance of differences between force and <P_2> were assessed with a two-tailed, paired Student's t-test: *p<0.05, **p<0.01, ns-not significant. Data from[1,2].

Supplementary References

1 Kampourakis, T., Sun, Y. B. & Irving, M. Myosin light chain phosphorylation enhances contraction of heart muscle via structural changes in both thick and thin filaments. *Proc Natl Acad Sci U S A*, doi:10.1073/pnas.1602776113 (2016).

2 Kampourakis, T., Zhang, X., Sun, Y. B. & Irving, M. Omecamtiv Mercabil and Blebbistatin modulate cardiac contractility by perturbing the regulatory state of the myosin filament. *J Physiol*, doi:10.1113/JP275050 (2017).
