# Supplementary material for: The regulatory light chain mediates inactivation of myosin motors during active shortening of cardiac muscle
Source: Nat Commun. 2021 Sep 6;12:5272. doi: 10.1038/s41467-021-25601-8 (PMC8421338; doi:10.1038/s41467-021-25601-8)
Supplement: Supplementary file 3 — Reporting Summary [file 41467_2021_25601_MOESM3_ESM.pdf]

nature research

Corresponding author(s): Thomas Kampourakis

Last updated by author(s): Aug 12, 2021

# Reporting Summary

Nature Research wishes to improve the reproducibility of the work that we publish. This form provides structure for consistency and transparency in reporting. For further information on Nature Research policies, see our Editorial Policies and the Editorial Policy Checklist.

## Statistics

For all statistical analyses, confirm that the following items are present in the figure legend, table legend, main text, or Methods section.

| n/a | Confirmed | |
|---|---|---|
| ☐ | ☒ | The exact sample size ($n$) for each experimental group/condition, given as a discrete number and unit of measurement |
| ☐ | ☒ | A statement on whether measurements were taken from distinct samples or whether the same sample was measured repeatedly |
| ☐ | ☒ | The statistical test(s) used AND whether they are one- or two-sided<br>*Only common tests should be described solely by name; describe more complex techniques in the Methods section.* |
| ☒ | ☐ | A description of all covariates tested |
| ☒ | ☐ | A description of any assumptions or corrections, such as tests of normality and adjustment for multiple comparisons |
| ☐ | ☒ | A full description of the statistical parameters including central tendency (e.g. means) or other basic estimates (e.g. regression coefficient) AND variation (e.g. standard deviation) or associated estimates of uncertainty (e.g. confidence intervals) |
| ☐ | ☒ | For null hypothesis testing, the test statistic (e.g. $F$, $t$, $r$) with confidence intervals, effect sizes, degrees of freedom and $P$ value noted<br>*Give P values as exact values whenever suitable.* |
| ☒ | ☐ | For Bayesian analysis, information on the choice of priors and Markov chain Monte Carlo settings |
| ☒ | ☐ | For hierarchical and complex designs, identification of the appropriate level for tests and full reporting of outcomes |
| ☐ | ☒ | Estimates of effect sizes (e.g. Cohen's $d$, Pearson's $r$), indicating how they were calculated |

*Our web collection on statistics for biologists contains articles on many of the points above.*

## Software and code

Policy information about availability of computer code

| | |
|---|---|
| Data collection | Dedicated program written in LabView 2014 (National Instruments) |
| Data analysis | Microsoft Excel 2014, Graphpad Prism 9, Pymol 2.4.1., MAXCHELATOR Software |

For manuscripts utilizing custom algorithms or software that are central to the research but not yet described in published literature, software must be made available to editors and reviewers. We strongly encourage code deposition in a community repository (e.g. GitHub). See the Nature Research guidelines for submitting code & software for further information.

## Data

Policy information about availability of data

All manuscripts must include a data availability statement. This statement should provide the following information, where applicable:

- Accession codes, unique identifiers, or web links for publicly available datasets
- A list of figures that have associated raw data
- A description of any restrictions on data availability

The data supporting the findings of the study are available in the article and its Supplementary Information. The source data for Figures 1, 2, 3 and 4, and Supplementary Figures 1, 2, 3, 4 and 5 are provided as a Source data file. All remaining data will be available from the corresponding author upon reasonable request. Source data are provided with this paper.

# Field-specific reporting

Please select the one below that is the best fit for your research. If you are not sure, read the appropriate sections before making your selection.

- [x] Life sciences
- [ ] Behavioural & social sciences
- [ ] Ecological, evolutionary & environmental sciences

For a reference copy of the document with all sections, see nature.com/documents/nr-reporting-summary-flat.pdf

# Life sciences study design

All studies must disclose on these points even when the disclosure is negative.

| | |
|---|---|
| Sample size | No sample size calculations were performed. The sample size used for each experiment was chose to be statistically adequate to describe and interpret the results while minimizing the number of animals used in the study (in accrodance with the 3Rs of animal welfare). |
| Data exclusions | No data were excluded from analysis |
| Replication | All experiments included in the paper were repeated multiple times (three to nine biological repeats; please see individual figure legends for details). All replicates are biological repeats (i.e. each measurement corresponds to a different biological preparation). All attempts at replicating the data were successful. |
| Randomization | Samples were allocated randomly. |
| Blinding | Not relevant to current study. Experiments are not based on comparison between control and experimental groups. |

# Reporting for specific materials, systems and methods

We require information from authors about some types of materials, experimental systems and methods used in many studies. Here, indicate whether each material, system or method listed is relevant to your study. If you are not sure if a list item applies to your research, read the appropriate section before selecting a response.

## Materials & experimental systems

| n/a | Involved in the study | |
|---|---|---|
| x | | Antibodies |
| x | | Eukaryotic cell lines |
| x | | Palaeontology and archaeology |
| | x | Animals and other organisms |
| x | | Human research participants |
| x | | Clinical data |
| x | | Dual use research of concern |

## Methods

| n/a | Involved in the study | |
|---|---|---|
| x | | ChIP-seq |
| x | | Flow cytometry |
| x | | MRI-based neuroimaging |

## Animals and other organisms

Policy information about studies involving animals; ARRIVE guidelines recommended for reporting animal research

| | |
|---|---|
| Laboratory animals | Wistar rats, male, 200-250g, 8 weeks old |
| Wild animals | No wild animals were used in this study. |
| Field-collected samples | No field-collected samples were used in this study. |
| Ethics oversight | All animals were treated in accordance with the guidelines approved by the UK Animal Scientific procedures Act (1986) and European Union Directive 2010/63/EU. All procedures were performed according to Schedule 1 of the UK Animal Scientific Procedure Act, 1986, which do not require ethical approval. All procedures complied with the relevant ethical regulations and were carried out in accordance with the guidelines of the Animal Welfare and Ethical Review Body (AWERB, King's College London). |

Note that full information on the approval of the study protocol must also be provided in the manuscript.
